# Resilience as a Protective Factor in Substance Use Prevention: A Narrative Review of Health Promotion Strategies Across Adolescence and Young Adulthood

**DOI:** 10.3390/healthcare14152252

**Published:** 2026-07-23

**Authors:** Donna L. Roberts

**Affiliations:** College of Arts & Sciences, Embry-Riddle Aeronautical University—Worldwide, Daytona Beach, FL 32114, USA; donna.roberts@erau.edu

**Keywords:** resilience, substance use prevention, addiction prevention, health promotion, adolescents, young adults, protective factors, public health

## Abstract

**Highlights:**

**What are the main findings?**

- Resilience is most useful for substance use prevention when it is understood as a multilevel developmental process supported by individual, relational, institutional, community, and cultural protective factors.
- Prevention strategies appear strongest when they integrate coping, connectedness, school or campus engagement, family support, and community-centered health promotion rather than relying on risk messaging alone.

**What are the implications of the main findings?**

- Substance use prevention should be embedded within broader public health and mental health promotion systems that strengthen supportive environments across adolescence and young adulthood.
- Future prevention research and practice should prioritize developmental precision, cultural responsiveness, and equity-aware multilevel intervention design.

**Abstract:**

Background/Objectives: Substance use disorders remain a significant public health concern, particularly when vulnerability emerges during adolescence and young adulthood. Although much of the literature has focused on treatment and recovery, prevention-oriented scholarship increasingly points to the value of strengths-based approaches that reduce risk by cultivating protective processes. This narrative review examines resilience as a protective factor in substance use prevention across adolescence and young adulthood, with particular attention to health promotion, developmental context, and multilevel prevention systems. Methods: A narrative review approach was used to synthesize interdisciplinary literature from psychology, public health, education, addiction studies, and prevention science. The review focused on peer-reviewed scholarship addressing resilience, protective factors, and prevention-related processes relevant to adolescent and young adult substance use. Literature was analyzed thematically across major domains including conceptualizations of resilience, developmental relevance, protective factors, prevention strategies, diverse and at-risk populations, and conceptual and methodological challenges. Results: The literature suggests that resilience is most useful for substance use prevention when conceptualized as a dynamic and ecological process rather than a fixed individual trait. Protective processes associated with lower substance use risk include emotional regulation, adaptive coping, family support, peer connectedness, school engagement, community cohesion, and culturally meaningful sources of identity and belonging. Prevention strategies appear most promising when they are multilevel, relational, developmentally responsive, and integrated with broader mental health and health promotion efforts. At the same time, the literature remains limited by definitional inconsistency, measurement variability, heterogeneous outcome indicators, a predominance of cross-sectional evidence, inconsistent or null protective effects for some factors and outcomes, and insufficiently contextualized uses of resilience. Conclusions: Resilience offers a valuable framework for substance use prevention across adolescence and young adulthood when it is grounded in developmental, ecological, and equity-aware perspectives. Prevention may be strengthened by moving beyond deficit-focused models and intentionally cultivating the supportive conditions under which young people can adapt, connect, and thrive without turning to substances as a means of coping.

## 1. Introduction

Substance use disorders remain a major public health concern in the United States and globally, with consequences that extend far beyond individual health outcomes to affect families, educational systems, workplaces, and communities [1,2,3,4,5]. The burden associated with harmful substance use includes increased morbidity and mortality, reduced psychosocial functioning, impaired educational and occupational attainment, and substantial downstream social and economic costs [1,2,3]. These concerns are especially pronounced when substance-related risk emerges during adolescence and young adulthood, developmental periods characterized by rapid change, experimentation, and increasing exposure to social and environmental pressures [2,3,4,5]. Although the harms associated with problematic substance use are well recognized, the pathways leading toward such outcomes are complex and are shaped by psychological, relational, and structural influences rather than by any single risk factor alone [1,2,3]. As a result, prevention efforts that address only isolated behaviors without considering broader developmental and contextual processes may be insufficient to reduce long-term vulnerability [1,2].

Within the substance use literature, scholarly attention has often centered on treatment, diagnosis, recovery, and relapse prevention, while comparatively less emphasis has been placed on primary and secondary prevention frameworks that seek to reduce the likelihood that harmful patterns develop in the first place [1,3,5]. This imbalance has practical consequences. When prevention is overshadowed by treatment-oriented discourse, opportunities to identify and strengthen protective processes earlier in the developmental trajectory may be missed [1,3]. In recent years, however, there has been growing recognition that prevention should not be confined to warning-based or deficit-focused models alone. Instead, substance use prevention may benefit from broader health promotion approaches that cultivate adaptive functioning, strengthen social supports, and reduce reliance on maladaptive coping strategies [1,2,5]. Such a shift is especially compatible with public health models that emphasize upstream influences, community context, and the interaction between individual behavior and social environment [1,2].

One concept that has gained increasing relevance in this regard is resilience. Although resilience has been defined in multiple ways across disciplines, it is commonly associated with the capacity to adapt positively under conditions of stress, adversity, or developmental challenge [6,7,8]. In the context of substance use prevention, resilience offers a particularly valuable lens because it redirects attention from pathology alone toward the processes that help individuals and communities withstand risk, recover from stress, and maintain healthier patterns of functioning [5,6,7,8]. This strengths-based perspective does not deny the importance of risk, trauma, or structural disadvantage; rather, it broadens the analytic frame to include the protective capacities and contextual supports that may interrupt or mitigate substance-related vulnerability [6,7,8]. By highlighting adaptive coping, supportive relationships, institutional belonging, and community resources, resilience-oriented perspectives may help prevention scholarship move beyond narrow behavioral explanations toward more integrative models of health promotion [5,6,7,8].

Resilience is especially relevant during adolescence and young adulthood because these developmental periods involve heightened sensitivity to stress and social influence, as well as important transitions in identity, autonomy, and belonging [3,4,5]. Adolescence is often marked by intensified peer salience, emotional volatility, exploratory behavior, and evolving cognitive control, all of which may shape vulnerability to substance-related experimentation and risk [4,5]. Young adulthood, although often treated as a more mature life phase, presents its own constellation of vulnerabilities, including educational pressure, employment uncertainty, relational instability, geographic mobility, and reduced external monitoring [3,4]. At the same time, both periods offer substantial opportunities for prevention because the developmental systems that support coping, decision making, social connection, and future orientation remain malleable [4,5,6]. A resilience-informed prevention framework is therefore well positioned to illuminate not only why some adolescents and young adults become vulnerable to harmful substance use, but also why others are able to navigate comparable pressures without following the same trajectory [3,4,5,6].

Importantly, resilience should not be understood as a simple matter of personal toughness or individual willpower. A growing body of scholarship has challenged overly individualistic interpretations by emphasizing that resilience is often relational, contextual, and ecological in nature [6,7,8,9,10]. Young people do not adapt in isolation; their capacity to cope effectively is shaped by family dynamics, peer norms, school or campus climate, community cohesion, access to support services, and broader structural conditions [7,8,9,10,11,12,13]. Understood in this way, resilience offers a useful organizing framework for substance use prevention across adolescence and young adulthood because it integrates individual-level processes such as emotional regulation and coping with relational, institutional, and community influences, encourages attention to diverse populations and the structural determinants of health, and reframes prevention as the active cultivation of capacities, environments, and relationships that reduce the appeal or necessity of substance use as a maladaptive coping mechanism [1,2,5,6,7,8,9,10,11,12,13,14,15,16]. This perspective is especially relevant to contemporary public health discourse, which increasingly emphasizes the integration of mental health promotion, social support, and prevention science [2,10].

The purpose of this narrative review is to synthesize and critically evaluate the literature on resilience as a protective factor in substance use prevention across adolescence and young adulthood. Specifically, this review examines how resilience has been conceptualized in relation to prevention, identifies key protective factors associated with reduced substance use risk, considers major health promotion and prevention strategies that appear to strengthen resilience, and addresses the relevance of these questions for diverse and at-risk populations. In addition, this review discusses conceptual and methodological limitations in the current literature and outlines priorities for future research. In concise terms, the review has three objectives: first, to clarify how resilience and its related protective factors should be conceptualized for substance use prevention; second, to synthesize, across the individual, relational, institutional, and community levels, the protective factors and the prevention and health promotion strategies that strengthen them throughout adolescence and young adulthood; and third, to appraise the strength and limitations of the underlying evidence and derive priorities for research and practice. By bringing together scholarship from psychology, public health, education, and related fields, this review evaluates the extent to which the available evidence supports conceptualizing resilience as a multilevel, developmental, and community-supported process rather than as an isolated individual trait, giving explicit attention to inconsistent findings, the strength of the underlying study designs, and the empirical basis for multilevel claims.

The specific contribution of this review is therefore not to restate that resilience is dynamic, ecological, or multilevel, since these claims are already well established in prevention science, but to integrate three elements that are usually treated separately. First, it spans adolescence and young adulthood within a single developmental framework rather than treating them in isolation. Second, it organizes both protective factors and prevention strategies along the same set of ecological levels, so that the two can be read as a coherent crosswalk from where protection resides to where prevention can act. Third, it foregrounds the strength and temporality of the underlying evidence when appraising each level. This integrative, evidence-weighted mapping across developmental stages and ecological levels, rather than any single conceptual assertion, is the review’s intended main thread.

## 2. Review Approach

This article was conducted as a narrative review designed to synthesize and interpret interdisciplinary literature on resilience as a protective factor in substance use prevention across adolescence and young adulthood [17,18]. A narrative review was selected because the literature relevant to this topic is conceptually diverse, methodologically heterogeneous, and distributed across several fields, including psychology, public health, addiction studies, education, community health, and prevention science [5,6,7,8,17,18]. Unlike a narrowly quantitative review, this approach allows for the integration of conceptual work, empirical studies, and applied prevention literature in order to clarify major themes, identify patterns across settings and populations, and highlight unresolved tensions or gaps in the field [17,18]. The goal of the review was therefore not to generate a pooled statistical estimate, but rather to provide a critical and constructive synthesis of how resilience is being used, understood, and operationalized within substance use prevention scholarship [17,18].

The literature search was designed to be broad enough to capture the interdisciplinary character of the field while remaining focused on the review’s core objectives. Searches were conducted across major scholarly databases relevant to behavioral health, social science, and public health research, including PubMed, PsycINFO, Scopus, and Web of Science, supplemented by backward and forward citation tracking of key articles and reviews. Search terms were developed around the central concepts of resilience and prevention and included combinations of terms such as “resilience,” “protective factors,” “substance use prevention,” “addiction prevention,” “health promotion,” “adolescents,” “young adults,” “community prevention,” and “school-based prevention” [17,18]. Additional searches were used where appropriate to identify relevant literature on associated constructs, including coping, social connectedness, self-efficacy, and developmental assets, when such constructs were clearly linked to resilience-oriented prevention frameworks. The primary database search was conducted and iteratively updated during manuscript preparation, covering the period January 2024–December 2024, with the final database update completed in December 2024. The search was not extended beyond this date, and the most recent sources included in the review were published in 2024; the small number of sources added after initial screening was completed were identified through targeted backward and forward citation tracking of key articles and reviews rather than through renewed database searching, and no publications dated after December 2024 were incorporated. The search was limited to English-language, peer-reviewed publications, and no earlier date restriction was applied, so that foundational theoretical contributions could be considered alongside recent prevention research. Titles and abstracts of identified records were screened for relevance, and the full texts of potentially eligible sources were then assessed against the criteria described below. The databases, search terms, and inclusion and exclusion criteria that guided the review are summarized in Table 1.

**Figure 1:**
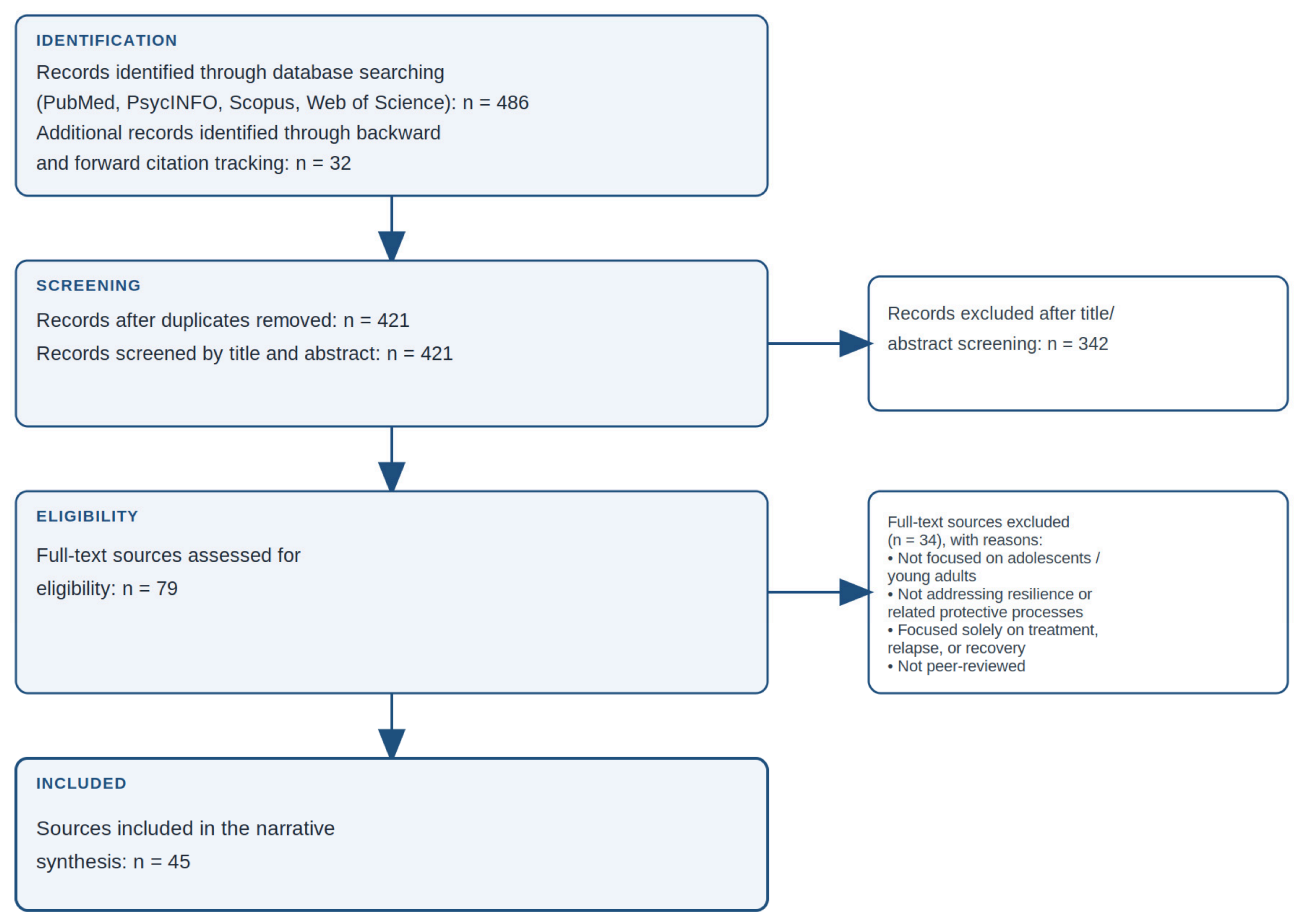
Flow of source identification, screening, and inclusion for the narrative review. Counts reflect the records handled at each stage of this purposive, single-author search; because the review was narrative rather than systematic, the diagram documents a purposive selection process rather than a PRISMA-style exhaustive search, and no formal quality appraisal or quantitative evidence grading was performed.

Screening and selection followed a staged process that is summarized in Figure 1. Records identified through database searching and citation tracking were first screened by title and abstract, and potentially relevant sources were then evaluated in full against the inclusion and exclusion criteria. Because narrative reviews aim for conceptual representativeness rather than exhaustive coverage, source selection was purposive: priority was given to conceptually foundational works, methodologically stronger empirical studies, and sources most directly informative for the review’s questions. This purposive strategy, rather than any scarcity of relevant publications, explains why a focused body of literature was retained from a much larger field. To avoid misinterpretation, it should be stated explicitly that this article is a narrative review, not a systematic review: the flow diagram in Figure 1 documents this purposive selection process for the sake of transparency and is not intended as a PRISMA-style record of an exhaustive search, and no formal quality appraisal or quantitative grading of the evidence was performed on the included sources. As a single-author review, screening, selection, and synthesis were conducted by one reviewer; consequently, formal inter-rater reliability could not be calculated, and this is acknowledged as a limitation in Section 8. To reduce the risk of selective or idiosyncratic interpretation, sources were re-examined iteratively against the review questions, an audit trail of included sources and their thematic coding was maintained, and the reference list was checked for balance across the individual, relational, institutional, and community levels that frame the review.

Priority was given to peer-reviewed articles and reviews that addressed resilience directly or examined closely related protective processes relevant to the prevention of substance misuse. The review focused primarily on literature concerning adolescents and young adults, with particular attention to studies and conceptual papers that addressed prevention before the onset of severe or entrenched substance-related disorder [1,2,3,4,5]. Sources were included when they contributed meaningfully to one or more of the following aims: clarifying the concept of resilience in prevention contexts; identifying protective factors associated with lower substance use risk; examining prevention or health promotion interventions intended to strengthen resilience; or addressing developmental, social, or contextual factors relevant to resilience-informed prevention [5,6,7,8,9,10,11,12,13,14,15,16]. Literature from multiple disciplines was intentionally included in order to reflect the fact that prevention and resilience are both cross-cutting constructs that cannot be adequately understood through a single disciplinary lens [17,18].

The review did not prioritize studies focused exclusively on treatment, relapse prevention, or recovery unless those studies offered conceptual or practical insights that could inform prevention-oriented thinking. Similarly, although broader discussions of mental health, trauma, stress, and social determinants were considered important, they were included only when they were directly relevant to resilience and substance use prevention rather than as standalone topics. Because resilience is often operationalized indirectly in the literature, some flexibility was necessary in determining inclusion. To keep this flexibility from becoming a route to selective citation, the inclusion of indirectly operationalized constructs required an explicit, stated link to protective processes relevant to substance use, and the review deliberately sought disconfirming as well as supportive evidence, an aim reflected in the treatment of null and mixed findings in Section 8. In such cases, studies examining developmental assets, coping resources, emotional regulation, relational support, belonging, or community connectedness were considered relevant if they clearly contributed to understanding the protective processes that may buffer against substance-related risk [6,7,8,9,10,11,12,13,14,15,16]. This approach allowed the review to remain conceptually focused while also accounting for the variability with which resilience is named and measured across fields [6,7,8,17,18].

The literature was synthesized thematically rather than chronologically. Consistent with established approaches to thematic synthesis, this involved repeated reading of the included sources to become familiar with their content, inductive coding of recurring concepts and findings, grouping of these codes into candidate themes, and refinement of the themes through constant comparison across sources and disciplines [17,18]. The single author carried out each of these steps and revisited earlier coding decisions as later sources were incorporated, so that the final thematic structure reflected iterative rather than one-pass interpretation. To weight the evidence, sources were appraised informally for methodological quality, with greater interpretive weight given to prospective, longitudinal, and intervention designs than to cross-sectional or single-informant self-report studies; no formal quantitative grading of evidence, such as a GRADE-style rating, was applied, consistent with the interpretive aims of a narrative review [17,18]. After reviewing the relevant sources, the material was organized around several major analytic domains: conceptualizations of resilience; developmental relevance across adolescence and young adulthood; protective factors associated with resilience and lower substance use risk; prevention and health promotion strategies that build resilience; considerations related to diverse and at-risk populations; and conceptual or methodological challenges in the literature. This thematic organization was selected because it best supported the review’s objective of building a coherent conceptual argument rather than presenting a study-by-study inventory [17,18]. Throughout the review, emphasis was placed on critically evaluating not only what the literature suggests about resilience and prevention, but also how the field’s underlying assumptions, definitions, and measurement practices shape those conclusions [6,7,8,17,18].

As a narrative review, this article does not claim exhaustive capture of every study related to resilience or substance use prevention. Instead, it aims to provide a transparent, analytically grounded synthesis of the most relevant themes and contributions in the literature, consistent with the goals of conceptual integration and scholarly interpretation [17,18]. This approach is particularly well suited to a field in which key constructs remain contested and where insights emerge from multiple types of scholarship rather than from one uniform evidentiary tradition [6,7,8,17,18]. The emphasis throughout is therefore on clarity of rationale, adequacy of search strategy, critical engagement with the literature, and the generation of meaningful directions for future research and public health practice [17,18].

## 3. Conceptualizing Resilience in Substance Use Prevention

Resilience is widely invoked in prevention scholarship, yet the concept remains variably defined across disciplines and research traditions [6,7,19,20,21,22]. In some accounts, resilience is described in broad developmental terms as positive adaptation despite adversity, whereas in others it is framed more specifically as effective functioning under conditions of stress, risk exposure, or trauma [6,7,19,21,22]. This definitional variability has contributed to the concept’s appeal, but it has also generated ambiguity in how resilience is interpreted, measured, and translated into prevention research and practice [7,20,21,22]. For substance use prevention, conceptual clarity is especially important because resilience is often used to explain why some adolescents and young adults do not follow harmful substance use trajectories despite exposure to multiple forms of risk [5,19,22]. Without greater precision, however, resilience can become a diffuse label applied to a wide range of positive outcomes without sufficient attention to the mechanisms through which those outcomes are achieved [7,21,22]. Table 2 summarizes major conceptualizations of resilience and their relevance to substance use prevention.

A central point in the resilience literature is that resilience should not be treated as a fixed trait or a simple personality characteristic [6,7,19,21,22]. Rather, resilience is more accurately understood as a dynamic process that is inferred from patterns of adaptation over time [22]. From this perspective, resilience is neither invulnerability nor the absence of distress; instead, it refers to relatively positive functioning in the context of adversity or risk exposure [7,19,22]. Masten’s influential formulation of resilience as “ordinary magic” emphasized that adaptive functioning often arises from normative developmental systems rather than rare or extraordinary individual qualities [6]. Similarly, Rutter argued that resilience is not a single attribute residing within the person, but an inferential concept shaped by developmental processes, environmental conditions, and the timing and nature of adversity [22]. These formulations are especially relevant to substance use prevention because they caution against interpreting resilience as a static inner strength and instead direct attention toward how protective systems operate across developmental contexts [6,19,22].

**Figure 2:**
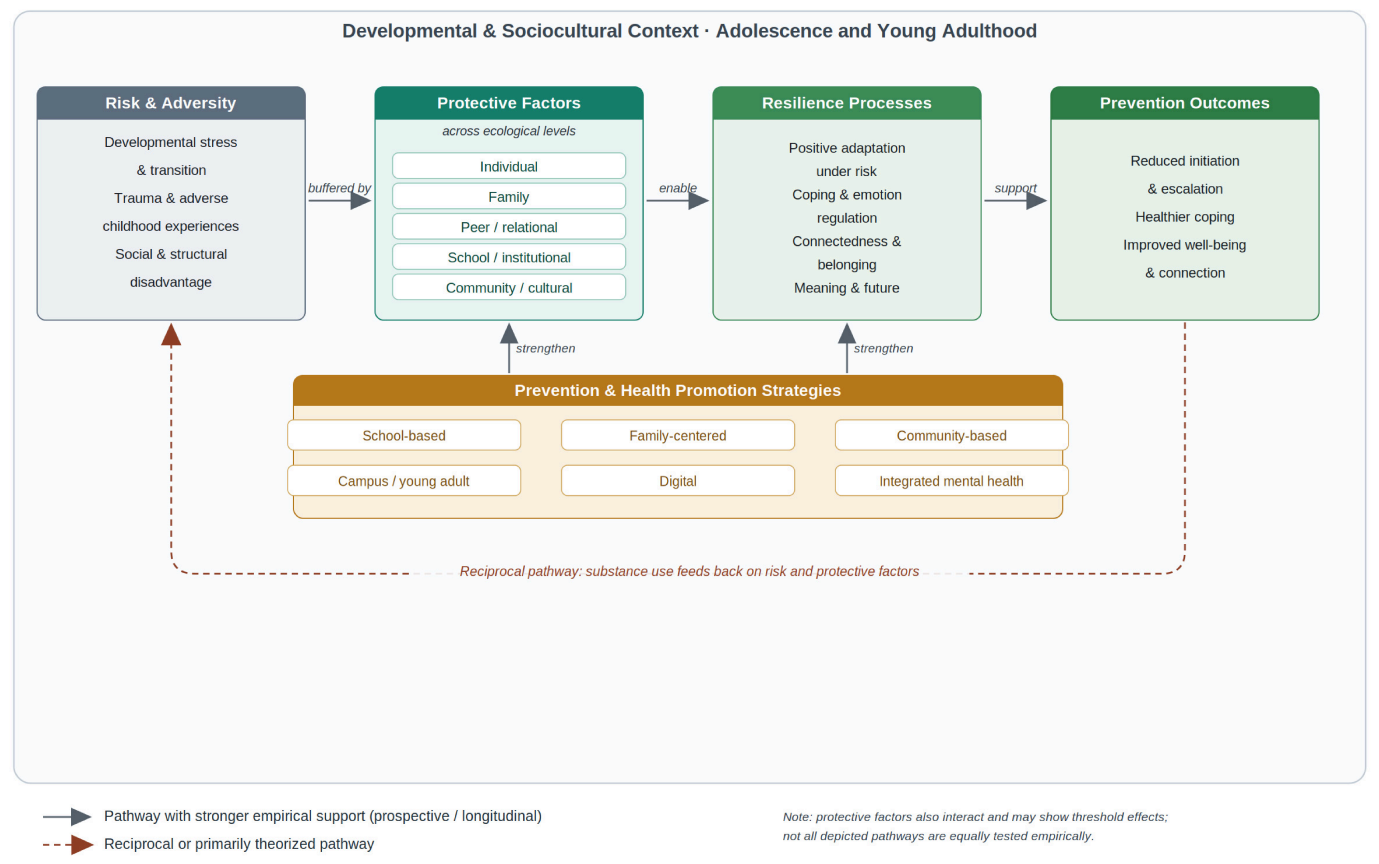
Conceptual model of resilience in substance use prevention. Risk and adversity are buffered by protective factors operating across individual, family, peer, school, and community or cultural levels, which in turn enable resilience processes that support prevention outcomes such as reduced initiation and escalation, healthier coping, and improved well-being. Prevention and health promotion strategies strengthen these protective factors and resilience processes, and the entire pathway is embedded within the developmental and sociocultural context of adolescence and young adulthood. The model is heuristic rather than strictly causal or purely linear: solid arrows denote pathways with comparatively stronger empirical support, the dashed arrow denotes a reciprocal and more theorized pathway in which substance use itself feeds back on risk and protective factors, and, as noted in the figure, protective factors are understood to interact and may show threshold effects rather than operating in a single unidirectional sequence.

Conceptual distinctions between resilience and related constructs are also necessary. Resilience overlaps with, but is not identical to, constructs such as coping, self-efficacy, competence, or emotional well-being [7,21,22]. Coping generally refers to strategies used to manage stress, whereas self-efficacy concerns beliefs about one’s capacity to act effectively; both may contribute to resilience, but neither alone is equivalent to resilience [7,21]. Likewise, the presence of psychological competence or positive affect does not by itself establish resilience unless those qualities are interpreted in relation to adversity or elevated risk [7,22]. Windle’s concept analysis emphasized that resilience involves processes of negotiation, adaptation, and management in the face of significant challenge, supported by assets and resources within both the individual and the environment [21]. This distinction matters for prevention scholarship because substance use research often measures resilience indirectly through related constructs, which can obscure whether investigators are examining resilience itself or only one of its possible components [7,21,22]. In summary, these constructs can be arranged hierarchically rather than treated as interchangeable: coping and self-efficacy are specific individual-level capacities; promotive and protective factors are the broader set of assets and resources, spanning the individual, relational, institutional, and community levels, that make positive adaptation more likely; and resilience is the higher-order process and outcome of adapting relatively well under adversity that these capacities and resources support. Table 2 and Figure 2 summarize and situate these relationships.

An ecological perspective further strengthens the conceptualization of resilience for prevention work. Fergus and Zimmerman argued that adolescent resilience is best understood through attention to assets and resources that support healthy development under conditions of risk [19]. Ungar similarly emphasized the social ecology of resilience, arguing that resilience depends not only on individuals’ capacities to navigate adversity, but also on the availability of meaningful resources and the ability of families, institutions, and communities to provide them in culturally relevant ways [20]. These perspectives are especially useful in substance use prevention because they shift the focus away from individual blame and toward the layered environments that shape vulnerability and protection [19,20]. Family support, peer relationships, school connectedness, mentoring, neighborhood context, and cultural identity can all influence whether young people experience stress as overwhelming, manageable, or growth-promoting [9,10,11,12,13,14,19,20]. In this sense, resilience is not merely what a young person “has,” but what a young person can access, mobilize, and sustain within a broader social ecology [20].

This ecological framing also helps clarify the relationship between resilience and promotive or protective factors. Resilience theory distinguishes between assets located within the individual and resources embedded in the environment, both of which may support positive development [19,23,24]. Promotive factors are especially important in this regard because they do not simply reduce exposure to risk; they actively enhance healthy functioning and may counteract, buffer, or interrupt pathways leading from adversity to negative outcomes [23,24]. Zimmerman and colleagues have argued that resilience-informed prevention should therefore focus not only on minimizing threats, but also on identifying and strengthening the promotive processes that enable adolescents to adapt successfully [23,24]. This is highly relevant to substance use prevention, where effective interventions may depend as much on building meaningful connection, self-regulation, and future orientation as on warning against the harms of substances themselves [1,12,13,23,24]. Such an approach aligns closely with public health and health promotion frameworks because it emphasizes the cultivation of strengths alongside the reduction of risk [1,19,23].

This relationship, however, must be specified carefully to avoid a circularity that has been noted in the resilience literature. If resilience were defined simply as the sum of the protective factors associated with lower substance use, then the claim that these factors predict resilience, and that resilience is protective, would be true by definition rather than by evidence. To avoid this, the present review treats resilience and its putative protective factors as analytically distinct: resilience refers to the process and outcome of relatively positive adaptation under conditions of adversity or risk, whereas protective factors, such as coping skills, self-efficacy, connectedness, or developmental assets, are the antecedent resources hypothesized to make that adaptation more likely. The substantive, testable question is thus whether these factors prospectively predict better adaptation and lower substance use under adversity, not whether they can be relabeled as resilience. Where the literature measures resilience only through such component constructs, this is treated in Section 8 as a measurement limitation rather than as confirmation of the framework. Consistent with this distinction, terminology is applied cautiously throughout the review: because much of the underlying literature is methodologically heterogeneous and often cross-sectional, the unqualified term “protective factor” is reserved, wherever possible, for associations supported by prospective or longitudinal evidence, and phrases such as “resilience-related protective processes,” “candidate protective mechanisms,” or “factors associated with lower substance use risk” are preferred where causal or temporal evidence remains limited.

In the context of substance use prevention, resilience is most useful when conceptualized as a multilevel protective process that reduces the likelihood that stress, adversity, or developmental challenge will lead to maladaptive coping through substance use [1,5,12,13,19,23,24]. This view does not imply that resilient adolescents are unaffected by adversity or that resilience guarantees the absence of substance use. Rather, it suggests that under comparable conditions of risk, some youth are better able to draw on internal assets and external supports that interrupt escalation toward harmful patterns of use [5,19,22]. Such supports may include emotional regulation, supportive caregiving, prosocial peer networks, school belonging, access to activities that foster purpose, and culturally meaningful sources of identity and connection [9,10,11,12,13,14,15,19,20,24]. Conceptualizing resilience in this way allows prevention researchers to move beyond binary distinctions between “at-risk” and “healthy” youth and toward a more developmentally sensitive understanding of how adaptive processes unfold over time [6,19,22].

Taken together, the resilience literature suggests that the concept has greatest value for substance use prevention when it is defined neither as a vague synonym for wellness nor as a fixed personal trait, but as a dynamic and contextually embedded process of adaptation [6,7,20,21,22]. Figure 2 presents a conceptual model that situates resilience within this multilevel process, linking risk and adversity, protective factors across ecological levels, resilience processes, prevention and health promotion strategies, and substance-related outcomes. It also provides a conceptual bridge to the next section of this review, which considers why adolescence and young adulthood are especially important developmental periods for examining how resilience operates in substance use prevention [4,5,19].

## 4. Developmental Relevance Across Adolescence and Young Adulthood

Adolescence represents a particularly important developmental window for substance use prevention because it combines heightened vulnerability with substantial opportunity for adaptive change [4,5,25,26]. For the purposes of this review, adolescence is treated as approximately ages 10 to 18 and young adulthood as approximately ages 18 to 25, extending into the late twenties in some accounts of emerging adulthood [3,4]; these boundaries are approximate and overlapping rather than fixed, reflecting the gradual and individually variable nature of the transition. Framed in terms of resilience, this same malleability is what gives prevention its leverage: the developmental features that heighten risk also make protective processes and preventive intervention capable of redirecting trajectories, so that resilience operates here as a preventive mechanism rather than as a fixed trait. This period is marked by rapid biological, cognitive, emotional, and social development, including increased sensitivity to reward, novelty, and peer evaluation, alongside still-maturing systems for self-regulation and long-term decision making [25,26]. These developmental dynamics help explain why experimentation and risk-taking often increase during adolescence, but they also underscore why adolescence should not be viewed only as a period of danger. The same plasticity that may heighten vulnerability also creates important opportunities for prevention, skill development, and positive redirection [5,25,26]. From a resilience perspective, adolescence is therefore best understood as a period in which developmental systems are highly responsive to both risk and protection [19,22,26].

One of the most salient developmental changes in adolescence is social reorientation. As adolescents spend more time with peers and become increasingly attentive to social status, acceptance, and belonging, peer contexts gain pronounced influence over behavior and self-concept [5,25,27]. Nelson and colleagues characterized this shift as a normative process in which adolescents increasingly develop relationships and patterns of social engagement outside the family system, a transition with important implications for both healthy development and psychopathology [27]. In the domain of substance use, this reorientation may elevate exposure to opportunities for experimentation and increase the salience of peer norms surrounding alcohol and other substances [5,25,27]. At the same time, it also means that peer connectedness, social attunement, mentoring, and prosocial group membership can serve as powerful protective influences when aligned with healthy developmental pathways [5,10,19,27]. Resilience-oriented prevention must therefore take peer processes seriously, not simply as sources of risk, but as potential channels of support, identity, and adaptive change.

Young adulthood extends these developmental considerations rather than replacing them. Arnett’s formulation of emerging adulthood emphasized that the late teens through the twenties constitute a distinct developmental period characterized by identity exploration, instability, self-focus, and transitions in work, education, residence, and relationships [4]. Stone and colleagues similarly noted that substance use problems often peak during emerging adulthood, a period in which individuals typically encounter increased autonomy together with reduced social control and greater exposure to novel environments [3]. For many young adults, these transitions are accompanied by new stressors, weaker day-to-day monitoring, and wider opportunities for both experimentation and escalation [3,4]. Yet young adulthood is also the period during which many individuals begin to “mature out” of heavier patterns of use as adult roles, changing social expectations, and greater behavioral control become more salient [5,28]. This makes young adulthood a critical stage for prevention and health promotion, not only because substance-related vulnerability may remain elevated, but because adaptive change is still unfolding and can be meaningfully shaped [3,4,28].

A developmental view of resilience helps explain why the same young person may be vulnerable in one context and protected in another. During adolescence, resilience may depend more heavily on family support, school climate, external structure, and access to caring adults who scaffold coping and decision making [9,11,12,13,19,24]. As youth move into young adulthood, resilience may increasingly involve the integration of internal capacities such as self-regulation, goal orientation, and future planning with evolving external supports such as peer networks, institutional belonging, and meaningful role commitments [3,4,15,16,24,28]. This does not mean that relational factors become less important with age; rather, the form of those relationships and the settings in which protection is anchored may shift across development [10,19,24]. Prevention efforts that ignore these developmental transitions risk treating adolescence and young adulthood as interchangeable periods, when in fact the mechanisms of both vulnerability and resilience may differ in important ways [3,4,5,19,24].

A further question is whether resilience during young adulthood represents a genuinely distinct phenomenon or simply the downstream continuation of capacities and supports established earlier in adolescence. Both continuity and discontinuity appear to operate. On one hand, protective assets such as self-regulation, school bonding, and supportive relationships tend to show developmental continuity, so that adolescents who are better resourced often enter young adulthood with advantages that continue to lower risk; in this sense, some young-adult resilience is carried forward rather than newly created [15,16,24]. On the other hand, young adulthood introduces developmental processes that are not reducible to adolescent functioning. The expansion of autonomy, the assumption of adult roles, and the reorganization of social networks create new configurations of risk and protection, and longitudinal work on drinking trajectories indicates that many individuals shift substantially during this period, including the widely observed pattern of “maturing out” of heavier use as adult roles and self-regulatory capacities consolidate [3,4,28]. Person-centered analyses that identify distinct trajectory classes across young adulthood suggest that these divergent pathways are not fully predicted by adolescent status alone, implying that resilience in young adulthood is partly continuous with, and partly distinct from, its adolescent antecedents [28]. For prevention, this distinction matters: strategies for young adults should not simply assume the persistence of adolescent protective structures but should also address the role transitions, identity commitments, and changing social ecologies that are specific to this stage [3,4,28].

Stated at the level of mechanism rather than context, the two stages differ in how risk and protection actually operate. In adolescence, the operative mechanisms are largely neurodevelopmental and externally scaffolded: still-maturing self-regulation together with heightened reward and peer sensitivity means that protection works chiefly by supplying external structure, monitoring, and supportive adults. In young adulthood, the operative mechanisms are more self-directed and identity-based: the negotiation of autonomy, the consolidation of adult roles, and the reorganization of social networks mean that protection works less through external scaffolding and more through internalized goals, role commitments, and self-selected relationships. Table 3 summarizes these contrasts, which imply that a strategy effective at one stage may require redesign, rather than simple extension, to remain effective at the other.

Taken together, the literature suggests that adolescence and young adulthood should be understood as linked but distinct periods within the developmental architecture of substance use prevention [3,4,5,25,26,27,28]. Adolescence is characterized by heightened plasticity, social sensitivity, and vulnerability to peer-mediated risk, whereas young adulthood is defined more by transition, identity consolidation, expanding autonomy, and the gradual emergence of adult roles [4,25,27,28]. In both periods, resilience is not a static personal trait but a shifting developmental process supported by changing configurations of internal capacities and external resources [19,22,24]. This developmental framing is essential for the present review because it clarifies why protective factors cannot be treated as uniform across age groups (Table 3). The next section therefore examines the specific protective factors associated with resilience and lower substance use risk across these developmental stages.

## 5. Protective Factors Associated with Resilience and Lower Substance Use Risk

Protective factors provide the most concrete way to understand how resilience operates in substance use prevention. Whereas resilience refers to the broader process of positive adaptation under adversity, protective and promotive factors help explain the mechanisms through which that adaptation becomes possible [19,23,24]. This distinction is important because resilience is not directly observed as a single attribute; rather, it is inferred from patterns of functioning that are supported by internal assets and external resources [7,19,22,23,24]. In prevention research, protective factors are especially valuable because they shift analysis beyond the identification of risk and toward the conditions that interrupt, buffer, or redirect maladaptive developmental trajectories [1,19,23,24]. For substance use prevention, the literature suggests that protective factors are best understood as multilevel and cumulative, operating across individual, family, peer, school, community, and cultural domains rather than within any one domain alone [1,2,12,13,19]. These multilevel resilience-related protective processes and candidate protective mechanisms are summarized in Table 4.

At the individual level, several personal capacities are consistently associated with lower substance use risk and are often conceptualized as resilience-related assets. These include emotional regulation, effective coping, self-efficacy, problem-solving ability, future orientation, and beliefs that support healthy decision making [1,2,19,23,24]. Such factors do not eliminate exposure to risk, but they may reduce the likelihood that stress, adversity, or social pressure will be managed through maladaptive coping strategies such as substance use [1,2,12,24]. Reviews of adolescent substance use have also identified optimism, self-control, mindfulness, strong beliefs against substance use, and the desire to preserve one’s health as factors associated with lower substance use risk, although much of this evidence is cross-sectional [2]. From a resilience perspective, these individual assets are most meaningful when understood not as isolated personality traits, but as capacities that develop through interaction with family, school, and community supports [19,20,23]. This framing is especially important because individual strengths are more likely to function protectively when they are reinforced by stable and supportive environments [19,24].

Family-level protective factors remain among the most consistently documented influences in substance use prevention. Hawkins and colleagues identified family management practices, parental bonding, and clear behavioral expectations as important protective processes in reducing alcohol and drug-related risk [1]. Subsequent work has continued to demonstrate that parental monitoring, emotional warmth, communication, and cohesive family relationships are associated with lower rates of adolescent substance use and later problem behaviors [2,9,12,13]. Resnick and colleagues’ landmark Add Health analysis found that parent-family connectedness functioned as a strong protective factor across multiple adolescent health-risk domains, including substance use [9]. These findings suggest that resilience is often scaffolded by family systems that provide both emotional security and behavioral structure [1,9,13]. Even as adolescents move toward greater autonomy, the presence of dependable caregiving and family support may continue to shape how they interpret stress, regulate emotion, and navigate risky contexts [9,19].

Peer and broader relational factors also play an important role, particularly during adolescence and the transition into young adulthood. Peer influence is often discussed primarily as a source of risk, but the resilience literature suggests that peer processes can be protective when they reinforce belonging, prosocial norms, and adaptive coping [5,10,19,27]. Supportive friendships, mentoring relationships, and social environments that reward healthy behavior may reduce vulnerability to substance use by increasing connectedness and offering alternative sources of identity, support, and status [10,19,24]. Cousijn and colleagues’ developmental account of adolescent resilience to addiction emphasized the importance of the social environment, arguing that social attunement can increase risk in some contexts but also facilitate normative de-escalation and adaptation in others [5]. This is consistent with broader connectedness scholarship showing that meaningful social bonds are central to adolescent well-being and may function as candidate protective mechanisms when youth are under stress [10]. Accordingly, substance use prevention should treat relational connectedness not merely as background context, but as a core component of resilience itself [5,10,19].

Schools and other youth-serving institutions represent another major domain of protection. A growing body of evidence indicates that school connectedness, engagement, and perceived membership are associated with lower substance use and broader positive developmental outcomes [9,10,11,12,13,15]. Resnick and colleagues identified school connectedness as protective against a wide range of adolescent health-risk behaviors [9], and Weatherson and colleagues later provided prospective evidence that increases in school connectedness were associated with lower likelihood of smoking, marijuana use, and binge drinking over time [11]. Research on school engagement likewise suggests that connection to school may function as a malleable target for prevention, with benefits that extend across adolescence and into later developmental stages [15]. These institutional protections may operate through multiple pathways, including strengthened belonging, exposure to supportive adults, structured opportunities for competence and participation, and reduced alienation from conventional systems of support [10,11,15]. For resilience-informed prevention, schools are therefore not merely sites of exposure or surveillance; they are potential protective ecosystems that can shape coping, connection, and long-term developmental direction [11,15,24].

Community and cultural factors further extend the protective architecture of resilience. Community cohesion, access to structured activities, supportive local norms, and the presence of prosocial adults can all contribute to lower substance use risk and healthier adaptation [1,2,10,12,14,20]. Cleveland and colleagues found that among several protective domains, community-level factors showed particularly strong associations with adolescent substance use outcomes [12]. At the same time, scholarship on diverse and historically marginalized populations has emphasized that culturally meaningful sources of protection, such as positive identity development, intergenerational ties, and community-recognized pathways to belonging and purpose, may be especially important [14,20]. Woods and colleagues’ review of substance use among American Indian/Alaskan Native youth, for example, highlighted the importance of connected relationships with prosocial adults and positive cultural identity as key protective themes [14]. These findings reinforce Ungar’s argument that resilience depends not only on an individual’s ability to navigate adversity, but also on the capacity of social environments to provide resources in ways that are accessible, meaningful, and culturally relevant [20]. In this sense, community and culture do not simply surround resilience; they help constitute it [14,20].

Taken together, the protective factors associated with lower substance use risk suggest that resilience should be conceptualized as a layered and cumulative process rather than as the sum of isolated variables [1,2,19,23,24]. Individual strengths such as self-regulation and future orientation matter, but they are more likely to function effectively when supported by family cohesion, healthy peer networks, school connectedness, and communities that provide opportunity, meaning, and belonging [9,10,11,12,13,14,15,19,20]. This cumulative perspective also helps explain why prevention effects may vary across settings and populations: the absence of one source of protection may be offset in part by another, whereas multiple simultaneous deficits may magnify vulnerability [1,19,24]. Importantly, this multilevel interpretation is not only conceptual but has been examined empirically. Studies that model protective factors across several ecological levels simultaneously provide the most direct test: Cleveland and colleagues, for example, assessed individual, peer, family, and community protection together and found that the relative contribution of each level differed, with community and family factors emerging as comparatively strong predictors of adolescent substance use [12]. Longitudinal and cross-national analyses of multiple risk and protective domains similarly show that protection is distributed across levels rather than concentrated in any single one [13], and long-term evaluations of family-focused prevention delivered through school and community systems have demonstrated durable reductions in substance-related outcomes years later [16,29]. These empirical examples, rather than conceptual preference alone, are the basis for treating protection as a multilevel process. For this reason, the most useful prevention frameworks are those that consider how protective factors interact across developmental and ecological levels rather than focusing exclusively on a single domain [19,23,24]. The next section therefore turns from the identification of protective factors to the practical question of how prevention and health promotion strategies can deliberately cultivate them.

## 6. Prevention and Health Promotion Strategies That Build Resilience

The prevention literature increasingly suggests that resilience is not only a descriptive framework, but also a practical target for intervention [23,24,29,30,31,32,33,34,35]. If resilience reflects the processes through which adolescents and young adults adapt positively under stress or adversity, then prevention strategies should aim to strengthen the promotive and protective conditions that support those processes over time [19,23,24]. This shifts prevention away from models that focus primarily on warning, surveillance, or problem avoidance and toward approaches that intentionally cultivate coping, connectedness, self-regulation, and supportive environments [19,23,24,35]. In this sense, resilience-building prevention is closely aligned with health promotion because it emphasizes the development of capacities and contexts that support healthier trajectories before substance-related problems become entrenched [19,23,24]. The intervention literature further suggests that the most promising strategies are those that address multiple ecological levels rather than concentrating narrowly on information delivery alone [29,30,32,35]. Representative resilience-building prevention strategies across these levels are summarized in Table 5.

School-based prevention remains one of the most important platforms for resilience-oriented substance use prevention because schools reach large numbers of adolescents during a developmentally formative period [11,15,30,35]. Evidence from integrated academic and health education suggests that school-based interventions may be more effective when they are embedded in broader developmental and educational processes rather than delivered as isolated substance-focused messages [35]. Melendez-Torres and colleagues argued that integrating academic and health education may help prevent substance use by strengthening engagement, social bonding, and school commitment alongside health knowledge and behavioral skills [35]. This is consistent with broader findings that school connectedness and engagement function as important protective processes in their own right [11,15]. From a resilience perspective, school-based prevention is most powerful when it treats schools not merely as venues for curriculum delivery, but as protective environments capable of strengthening belonging, competence, supportive adult relationships, and adaptive coping [11,15,24,35]. Such an orientation helps explain why interventions that support the overall developmental climate of the school may have more durable effects than programs focused only on short-term risk messaging [15,35].

Family-centered prevention strategies are similarly important because they target one of the most durable and influential contexts of adolescent development [1,9,12,13,29,30]. Systematic review evidence indicates that parent-based interventions can reduce or prevent adolescent substance use when they strengthen communication, monitoring, rule-setting, and relational quality [29]. Kuntsche and Kuntsche concluded that parent-based approaches are especially relevant because they address family processes that shape how adolescents interpret stress, regulate behavior, and respond to opportunities for substance use [29]. Combined student- and parent-based programs may be particularly promising, as Newton and colleagues found that interventions spanning both school and family domains offer a broader platform for prevention than either setting alone [30]. These multicomponent approaches reflect a resilience-informed model because they do not rely exclusively on adolescents’ individual decision making; instead, they recognize that effective coping and behavioral regulation are supported by family structure, parental engagement, and consistent relational reinforcement [19,24,29,30]. For substance use prevention, this means that strengthening resilience often begins not with the individual adolescent alone, but with the relational systems that organize everyday developmental experience [19,29].

Community-based prevention strategies extend this multilevel approach by mobilizing local coalitions, shared norms, and community resources to reduce substance-related risk and enhance protective supports [1,20,32]. The Communities That Care prevention system is one of the clearest examples of a community-level approach that aligns with resilience theory because it emphasizes the assessment of local risk and protection, the coordinated implementation of evidence-based prevention programs, and the strengthening of community prevention infrastructure [32]. Van Horn and colleagues found that the Communities That Care system was associated with more favorable cross-sectional profiles of adolescent substance use and delinquency, suggesting that prevention can be strengthened when communities organize around shared protective goals [32]. Community-based strategies are particularly important from a resilience perspective because they broaden prevention beyond individual or family behavior and situate it within local opportunity structures, adult support networks, and collective norms [20,32]. They also align closely with the Special Issue’s emphasis on community-centered public health approaches to preventing substance-related harm. More broadly, these approaches underscore that resilience is sustained not only by what young people know or believe, but also by what communities make possible, accessible, and culturally meaningful [20,32].

In young adult populations, particularly in colleges and universities, prevention must respond to a distinct developmental landscape shaped by autonomy, identity exploration, peer influence, and reduced external monitoring [3,4,33]. Dennhardt and Murphy’s review of college student drug use emphasized that effective prevention in this population must account for the developmental and social realities of campus life rather than assuming that strategies designed for younger adolescents can simply be extended upward without modification [33]. It is important to note, however, that the evidence on young-adult prevention is itself skewed toward college and university samples, which represent only a portion of this age group. Young adults who are employed, unemployed, involved with the justice system, unstably housed, or living in rural areas are markedly under-represented in this literature, so campus-based findings should not be assumed to generalize to non-college young adults; extending resilience-informed prevention to these populations is an important priority, discussed further in Section 7 and Section 10. This is important because many emerging adults encounter environments in which experimentation is normalized and where stress, social pressure, and mental health needs intersect in complex ways [3,4,33]. Resilience-informed prevention in these settings may therefore be most effective when integrated into broader student well-being efforts that promote belonging, coping, help-seeking, and connection to meaningful campus roles and supports [10,15,33]. Rather than focusing exclusively on punitive messaging or rule enforcement, such approaches frame prevention as part of a healthier developmental transition into adulthood [3,4,33]. This interpretation is especially consistent with the present review’s argument that resilience is a developmental and ecological process rather than a fixed personal trait.

Digital and technology-mediated interventions represent another increasingly important domain of prevention, particularly for adolescents and young adults who may face barriers to in-person support or who are already accustomed to digital modes of communication and learning [31]. Stand-alone digital interventions have attracted attention because they may improve access to prevention services, increase privacy, and provide scalable tools for psychoeducation, self-monitoring, and coping support [31]. Aneni and colleagues’ systematic review and meta-analysis of stand-alone digital interventions for adolescent substance use prevention suggests that these approaches hold promise, although their effects depend on program design, engagement, and the quality of intervention content [31]. This promise should be read cautiously. The underlying digital trials differ widely in design and content, commonly report short follow-up periods, vary considerably in engagement and retention, and use heterogeneous outcome measures, so their comparative effectiveness and durability relative to in-person approaches remain uncertain [31]. From a resilience perspective, digital tools are unlikely to replace the importance of relationships, schools, families, or communities, but they may serve as useful supplements that reinforce coping skills, strengthen self-reflection, and provide timely support in moments of stress or vulnerability [19,24,31]. Their value may be especially high when they are integrated into broader prevention ecologies rather than delivered as isolated technological solutions [31]. Accordingly, digital prevention should be understood as a potentially important extension of resilience-building strategy, but not as a substitute for the social and environmental foundations of resilience itself [20,31].

Integrated mental health and substance use prevention approaches may be particularly important because many of the vulnerabilities associated with substance use are intertwined with stress, anxiety, depression, trauma exposure, and social disconnection [5,10,34]. Dunne and colleagues’ review of youth engagement strategies in mental health and substance use interventions highlights the importance of involving young people meaningfully in the design and implementation of prevention efforts [34]. This is highly compatible with resilience-informed prevention because meaningful engagement can strengthen agency, relevance, trust, and connection, all of which are central to adaptive functioning in adolescence and young adulthood [19,24,34]. Likewise, integrated approaches that address both emotional well-being and substance use are better positioned to reduce the appeal of substances as tools for self-medication or social coping [5,10,34]. Such interventions also reflect a public health orientation by treating substance use not as an isolated behavior problem, but as part of a broader ecosystem of developmental stress, support, and adaptation [10,34,35]. In this way, the most effective prevention may be that which strengthens resilience by promoting mental health, social connection, and meaningful participation at the same time [10,24,34].

Overall, the prevention literature suggests that resilience-building strategies are strongest when they are multilevel, relational, and developmentally responsive [23,24,29,30,31,32,33,34,35]. School-based, family-centered, community-wide, campus-based, digitally supported, and youth-engaged interventions each contribute something important, but their effectiveness is likely to be greatest when they are understood as complementary rather than competing approaches [29,30,31,32,33,34,35]. These comparative characterizations, including the description of some strategies as more promising or better supported than others, rest on the presence, consistency, and design strength of the available evidence rather than on pooled effect sizes, and the review does not attempt a formal quantitative comparison of intervention outcomes. The evidence base is also uneven: it is more established and more often longitudinal for school- and family-based approaches than for the newer community-scaled, campus, and digital strategies, for which follow-up periods are frequently shorter and study designs more heterogeneous [29,31,32,35]. What unites these intervention models is their emphasis on strengthening the protective conditions that allow adolescents and young adults to cope more effectively, remain connected to supportive systems, and pursue healthier pathways under stress [19,23,24]. This reinforces the core argument of the present review: resilience in substance use prevention is not simply a matter of personal strength, but a function of how individuals, relationships, institutions, and communities interact to support positive adaptation [19,20,23,24]. The next section turns to how these resilience-based prevention principles apply in diverse and at-risk populations, where the distribution of protective resources and opportunities is often deeply unequal.

## 7. Resilience and Prevention in Diverse and At-Risk Populations

Resilience-based prevention cannot be understood adequately without attention to the diverse social, cultural, and structural contexts in which substance use risk emerges [14,20,24,36]. Adolescents and young adults do not encounter risk or protection on equal terms. Exposure to adversity, access to supportive relationships, institutional responsiveness, community resources, and the cultural relevance of prevention efforts all vary across populations and settings [14,20,24]. As a result, prevention strategies that appear effective in one context may have limited relevance in another if they fail to account for differences in lived experience, social position, or structural constraint [20,36]. This is particularly important for resilience-oriented work because the concept can be misused if it is framed as a universal personal resource while ignoring the unequal distribution of opportunity, safety, and support [20,22,36]. A more credible resilience framework therefore requires population-sensitive analysis and an explicit recognition that adaptation unfolds within conditions that are socially and structurally patterned [20,36].

To keep this discussion from becoming a simple list of vulnerable groups, it is useful to organize these considerations along two dimensions. The first distinguishes distal, structural determinants, such as poverty, racism, and rurality, which shape the distribution of both risk and access to protective resources, from more proximal cultural and relational processes, such as stigma, cultural identity, community connectedness, and trust in institutions, through which those structural conditions are experienced and either buffered or amplified. The second dimension concerns whether the mechanisms of resilience themselves differ across groups or whether it is mainly their availability that differs. The evidence suggests that both patterns occur. For some populations, distinctive protective mechanisms appear especially salient, such as cultural identity, enculturation, and community ownership for Indigenous and ethnic-minority youth [14,37,38,39], or dense informal kinship and community ties in rural settings [40]; for others, the core mechanisms may resemble those in the general population but are less accessible because of structural constraint [20,36]. Direct, well-powered comparisons of resilience mechanisms across these groups remain scarce, however, so several of these contrasts are best treated as hypotheses for targeted research rather than as settled conclusions.

For youth exposed to trauma and chronic adversity, resilience-based prevention is especially important but must be grounded in an understanding of cumulative stress exposure [22,24,41]. Adverse childhood experiences (ACEs), including abuse, neglect, and household dysfunction, are strongly associated with earlier initiation of alcohol use and greater substance-related vulnerability during adolescence [41]. Dube and colleagues found a graded relationship between ACE exposure and both ever drinking and earlier alcohol initiation, highlighting how childhood adversity can shape substance-related risk long before a disorder is established [41]. These findings underscore that prevention for trauma-exposed youth cannot rest solely on generic messages about refusal or risk avoidance. Instead, effective prevention must recognize that substance use may emerge within broader patterns of dysregulated stress response, emotional burden, and disrupted relational safety [22,24,41]. From a resilience perspective, this means that trauma-informed prevention should prioritize emotional regulation, relational trust, predictability, and supportive environments capable of interrupting maladaptive coping trajectories before they become entrenched [22,24,41].

Socially marginalized and underserved populations require similar attention to structural context. Economic precarity, discrimination, stigma, exclusion, and uneven access to supportive institutions can all influence both substance use risk and the availability of protective resources [14,20,36]. Cunningham and Saleh’s review of structural stigma, racism, and sexism studies in substance use and mental health reinforces the point that broader structural determinants are relevant to substance-related outcomes and cannot be reduced to individual choice alone [36]. This perspective is important for prevention because it challenges approaches that place responsibility entirely on young people while overlooking the policies, institutional practices, and social arrangements that shape exposure to stress and access to care [20,36]. It also aligns with Ungar’s social-ecological account of resilience, which emphasizes that resilience depends not only on individual navigation, but also on whether communities and systems provide meaningful resources in accessible and culturally valid forms [20]. Consequently, resilience-informed prevention in underserved populations must be equity-aware, which means strengthening supports while also recognizing and, where possible, addressing the structural conditions that constrain adaptive development [20,36].

Rural, remote, and under-resourced communities illustrate this issue clearly. Such communities may face distinctive barriers related to geography, transportation, service availability, confidentiality, and reduced access to prevention infrastructure, yet they may also possess protective strengths rooted in close social ties, local identity, and informal support networks [20,40]. Rhew and colleagues found that patterns of substance use risk and protection differed across rural youth contexts, indicating that “rural” should not be treated as a single, uniform category [40]. These findings caution against assuming that prevention models developed in urban or suburban settings will translate seamlessly into rural communities without adaptation [40]. At the same time, they support a resilience perspective in which local context matters profoundly, both in relation to risk exposure and in relation to the resources that can be mobilized to support prevention [20,40]. For public health practice, this suggests that prevention in rural and remote settings should build on locally available strengths while also acknowledging the resource limitations that may intensify vulnerability [20,40].

Cultural responsiveness is equally central to resilience-informed prevention, particularly for minority and Indigenous youth populations [14,20,37,38,39]. Lauricella and colleagues’ systematic review of culturally grounded prevention for minority youth populations found that many such interventions were developed from the “ground up,” using collaborative and community-informed approaches rather than merely adapting preexisting generic models [37]. This distinction is important because culturally grounded prevention is not limited to surface-level representation; it seeks to build prevention around the values, norms, worldviews, and lived experiences of the communities being served [37,39]. Hecht and colleagues’ evaluation of the keepin’ it R.E.A.L. curriculum demonstrated the importance of culturally grounded substance use prevention for ethnically diverse urban middle-school youth, while Okamoto and colleagues described similar logic in the development of culturally grounded drug prevention interventions for Indigenous youth populations [38,39]. These studies are highly relevant to resilience because they show that protective processes are not culturally neutral. The meaning of support, resistance, identity, belonging, and coping may vary across communities, and prevention is more likely to be effective when these meanings are treated as foundational rather than peripheral [20,37,38,39]. For diverse youth populations, culturally grounded prevention may therefore function not only as a matter of program relevance, but also as a mechanism for strengthening resilience through recognition, fit, and community ownership [37,38,39].

Taken together, the literature suggests that resilience-based prevention is most meaningful when it is context-sensitive, culturally responsive, and attentive to inequity [14,20,24,36,37,38,39,40,41]. Trauma exposure, social marginalization, rural isolation, and cultural mismatch can all weaken the conditions under which resilience develops, even when individual strengths are present [20,36,40,41]. Conversely, prevention efforts that recognize structural barriers, build from community strengths, and reflect the cultural realities of the populations they serve are more likely to generate the forms of belonging, agency, and adaptive coping that resilience theory describes [20,24,37,38,39]. This is especially important for a public health approach to substance use prevention because it avoids reducing prevention to individual behavior management alone. Instead, it situates prevention within the broader social ecology of young people’s lives, where protection is created not only through personal skill, but also through equitable access to meaningful support, cultural validation, and safe developmental opportunities [20,36]. The next section turns to the conceptual and methodological challenges that continue to complicate the resilience and prevention literature.

## 8. Conceptual and Methodological Challenges in the Literature

Despite the promise of resilience-oriented prevention scholarship, the literature remains marked by several conceptual and methodological challenges that complicate interpretation and limit stronger conclusions [7,17,18,21,22,42,43]. One of the most persistent problems is the lack of consistency in how resilience is defined. Across studies, resilience may be treated as a trait, an outcome, a process, a cluster of protective factors, or a general synonym for positive functioning [7,21,22]. This variability weakens comparability across studies and makes it difficult to determine whether researchers are examining the same construct or only adjacent phenomena such as coping, competence, or well-being [7,21,22]. For prevention scholarship, this matters because the interpretation of resilience directly shapes how interventions are designed, how outcomes are measured, and how findings are translated into practice [19,23,24]. When the construct itself is unstable, the resulting literature may appear more coherent than it actually is [7,22].

Measurement presents a related challenge. Windle and colleagues’ methodological review of resilience measurement scales concluded that resilience instruments vary substantially in their theoretical assumptions, psychometric quality, and suitability for different populations [42]. More recently, Ballard and colleagues’ scoping review of adolescent resilience self-report scales similarly found considerable variation in the conceptual basis and adequacy of commonly used measures [43]. This is especially relevant to the present topic because many studies involving adolescents and young adults rely on self-report instruments that do not always distinguish clearly between resilience as adaptive functioning under adversity and broader indicators of psychological adjustment or social competence [42,43]. As a result, some findings may reflect the measurement of overlapping constructs rather than resilience per se. For substance use prevention research, this means that claims about resilience should be interpreted cautiously unless investigators specify how adversity, adaptation, and protective processes are being operationalized within their designs [7,21,22,42,43].

A further limitation concerns heterogeneity in the substance-related outcomes used across studies. Some studies assess lifetime use, others focus on recent use, initiation, binge patterns, frequency, problematic use, consequences, or attitudes toward substances [1,2,3,11,12,13,14,15,16]. These are not interchangeable outcomes, and they do not necessarily reflect the same developmental process or level of severity [1,2,3]. A protective factor associated with delayed initiation may not function in the same way for preventing escalation, hazardous use, or substance-related harms once use has begun [1,3,12]. Likewise, resilience-oriented interventions may influence some outcomes, such as coping motives or school engagement, before measurable changes appear in substance use frequency itself [15,16,35]. The diversity of outcomes in the literature therefore makes synthesis more difficult and can create the misleading impression that studies are directly comparable when they are in fact addressing quite different questions [1,2,3,17,18].

Because the field is inconsistent in exactly these respects, the present synthesis sought to keep measurement and outcome distinctions visible rather than collapsing them. Where possible, findings were interpreted as outcome specific, distinguishing initiation from escalation, frequency, binge or hazardous patterns, and substance-related problems, and noting when a protective association was demonstrated for one outcome but not others. Similarly, the review flagged whether resilience was assessed directly, through a dedicated resilience measure, or only indirectly through related constructs, since these support different inferential claims. The summary tables necessarily aggregate at the level of broad constructs and strategy types and should therefore be read together with these narrative qualifications rather than as evidence that the underlying measures and outcomes are equivalent.

A related and underemphasized issue is that protective effects are not observed uniformly across studies. Resilience-related factors that appear protective in one sample, for one substance, or at one developmental stage sometimes show weak, non-significant, or even reversed associations in others, and the present review, like much of the strengths-based literature, has tended to foreground supportive findings. Several considerations help explain this inconsistency rather than explain it away. First, resilience theory itself distinguishes among compensatory, protective, and challenge models, in which a factor may exert a direct main effect, may buffer risk only under high adversity, or may matter only within a certain range of exposure; the buffering (interaction) effects that most precisely correspond to the term “protective” are statistically demanding and are detected less consistently than main effects [19]. Second, whether a factor operates protectively is frequently conditional on context, dose, timing, gender, and the specific outcome examined, so that heterogeneity across studies is expected rather than anomalous [1,3,12]. Third, publication and reporting practices, together with the purposive selection typical of narrative reviews, likely bias the visible literature toward positive results, meaning that the apparent consistency of protective effects may be overstated [17,18]. Acknowledging null and mixed findings is not a concession that resilience is unimportant; rather, it clarifies that the relevant empirical question is not whether resilience is protective in general, but under what conditions, for whom, and for which outcomes particular protective processes operate [19,22,24].

The predominance of cross-sectional designs and self-report data further constrains causal inference. While the literature contains important longitudinal and prevention studies [11,13,15,16], many resilience and substance use studies remain correlational, which limits confidence about temporal direction and mechanism [17,18,22]. It is often unclear whether resilience-related factors reduce substance use risk, whether substance use erodes protective functioning, or whether both are shaped by other unmeasured variables operating simultaneously [3,22]. This distinction is more than methodological. Strictly, describing a factor as “protective” implies a temporal, prospective relationship in which the factor precedes and predicts lower subsequent risk, whereas concurrent associations cannot establish that precedence [19,22]. The strongest claims in this review therefore rest on the subset of prospective and longitudinal studies rather than on cross-sectional correlations: prospective evidence that increases in school connectedness predict lower later substance use [11], longitudinal analyses of risk and protective factors across adolescence [12,13], long-term follow-up of school-engagement effects [15], and fifteen-year outcomes of a family-focused prevention trial [16] provide firmer ground for inferring protection than do concurrent designs. Foregrounding this longitudinal evidence, and interpreting cross-sectional findings as suggestive rather than confirmatory, is essential to using the label “protective” responsibly. Self-report data introduce additional complexity. Winters and colleagues highlighted longstanding concerns about the validity of adolescent self-reported alcohol and other drug involvement [44], and Harris and colleagues showed that inconsistencies in adolescent self-reported drug use are not random and may affect outcome and performance measurement in meaningful ways [45]. These concerns do not invalidate self-report methods, which remain central to much prevention research, but they do reinforce the need for caution, triangulation, and stronger methodological design wherever possible [44,45]. Greater use of longitudinal studies, mixed-method approaches, and validated measurement strategies would strengthen the field considerably [15,16,42,43,44,45].

A final conceptual challenge is the risk of overindividualizing resilience. When resilience is framed too narrowly as personal toughness, grit, or the capacity to “overcome” adversity, prevention discourse may obscure the structural conditions that make adaptation more difficult for some youth than for others [20,22,36]. This is especially problematic in substance use prevention, where social determinants, stigma, discrimination, poverty, and unequal institutional support can shape both exposure to risk and access to protection [20,36]. A resilience framework that ignores these realities may inadvertently shift responsibility onto individuals while leaving systems and environments analytically underexamined [20,22,36]. For this reason, the most useful versions of resilience theory are those that retain a social-ecological orientation and treat protective processes as distributed across persons, relationships, institutions, and broader structural contexts [19,20,24]. This point is not merely theoretical; it directly affects how prevention priorities are defined and which interventions are viewed as legitimate or sufficient [20,23,24,36].

Taken together, these challenges suggest that the resilience and substance use prevention literature is promising but still conceptually uneven and methodologically mixed [7,17,18,21,22,42,43,44,45]. The field has generated important insights into protective processes and prevention possibilities, yet stronger progress will require clearer definitions, more consistent measurement, more developmentally precise outcomes, and research designs better suited to testing mechanisms and change over time [17,18,42,43]. It will also require continued resistance to reductive interpretations of resilience that detach it from context and inequity [20,36]. A further limitation applies to the present review itself: as a single-author narrative synthesis, it was not subject to independent double-screening or dual coding, and its purposive selection may under-represent null or contrary findings, so its conclusions should be weighed accordingly [17,18]. These limitations do not weaken the value of resilience as a prevention framework; rather, they define the conditions under which it can be used more rigorously and responsibly. The next section draws these themes together in a broader discussion of what the literature suggests about the role of resilience in public health approaches to substance use prevention.

## 9. Discussion

This narrative review suggests that resilience provides a valuable framework for substance use prevention when it is understood as a developmental, ecological, and health-promoting process rather than as a fixed individual trait [6,19,20,22,23,24]. Across the literature reviewed, the pattern that recurs most often, though not uniformly and more consistently in prospective than in cross-sectional studies, is that resilience-related protection emerges through the interaction of multiple factors, including emotional regulation, adaptive coping, family cohesion, peer connectedness, school engagement, community resources, and cultural fit [1,5,9,10,11,12,13,14,15,19,24]. As Section 8 notes, individual protective effects are sometimes inconsistent or absent, and the multilevel reading advanced here is therefore best understood as an inference from the balance of the stronger evidence, including studies that have tested protection across several ecological levels simultaneously [12,13,16,32], rather than as a uniform empirical regularity. These findings support the view that substance use prevention is most effective when it does not isolate behavior from context, but instead situates risk and adaptation within the broader systems that shape adolescent and young adult development [3,4,5,19,20]. The review also indicates that resilience is especially relevant during adolescence and young adulthood because these are periods of heightened plasticity, social transition, and vulnerability to both maladaptive coping and positive developmental redirection [4,25,26,27,28]. Taken together, the literature supports resilience not as a replacement for prevention science, but as an integrative lens that can help organize its developmental, relational, and public health dimensions [19,23,24].

A major conceptual contribution of resilience-based prevention is that it broadens the field beyond deficit-oriented models focused solely on risk exposure, pathology, or behavioral control [7,8,19,23,24]. Risk and harm remain essential concerns, but the present review suggests that prevention may be strengthened when it also attends to the promotive conditions that enable young people to adapt effectively under stress [19,23,24]. This strengths-based orientation is especially important in substance use prevention because it avoids equating prevention exclusively with abstinence messaging or disciplinary response. Instead, it reframes prevention as the cultivation of capacities and conditions that reduce the need for substances as tools of coping, self-medication, or social positioning [5,19,24,34]. In this respect, resilience aligns closely with health promotion by emphasizing not only what young people should avoid, but also what they need in order to flourish [10,23,24]. This may be one reason resilience-oriented frameworks hold particular promise for adolescents and emerging adults, who often respond more constructively to interventions that affirm agency, belonging, and development than to approaches based only on fear or control [5,10,24,34]. This is also where the present review’s specific contribution lies: not in cataloguing protective factors, but in aligning them, level by level and across both adolescence and young adulthood, with the prevention strategies meant to strengthen them, and in appraising the strength and temporality of the evidence for each alignment.

The review also reinforces the ecological character of resilience. The most persuasive literature does not locate resilience exclusively within the individual, but instead treats it as a process supported by relationships, institutions, communities, and cultural systems [19,20,24], which implies that prevention efforts focused only on individual knowledge or refusal skills may miss a large portion of the social ecology in which substance use decisions actually unfold [1,9,10,11,12,13,14,15,29,30,31,32,33,34,35]. The intervention literature reviewed here supports this interpretation: school-based, family-centered, community-level, digitally supported, and youth-engaged approaches appear most promising when they function as complementary parts of broader prevention ecologies rather than as isolated programmatic fixes [29,30,31,32,33,34,35].

From a public health standpoint, these findings suggest that effective substance use prevention should be integrated into systems that promote connectedness, mental health, developmental support, and social opportunity [1,2,5,10,23,24]. Prevention cannot be treated as a narrow add-on delivered only after risk becomes visible. Instead, it should be embedded in the ordinary developmental settings through which adolescents and young adults move, including families, schools, colleges, peer networks, health systems, and community organizations [10,11,15,29,30,31,32,33,34,35]. Such integration is especially important because many of the vulnerabilities associated with substance use are intertwined with loneliness, anxiety, trauma, social exclusion, and a lack of meaningful adult or institutional support [5,10,34,41]. Public health prevention efforts may therefore benefit from shifting from a predominantly reactive model to one that cultivates the everyday protective processes that resilience theory highlights [19,23,24]. In practice, this means designing prevention systems that support coping, engagement, belonging, and culturally meaningful connection at the same time that they reduce exposure to specific substance-related risks [20,24,29,30,31,32,33,34,35].

Educational institutions deserve particular attention within this discussion because they are among the most influential developmental environments for both adolescents and emerging adults [9,10,11,15,33,35]. Schools can foster resilience by strengthening school connectedness, relational trust, competence, and access to caring adults, while colleges and universities can do so by embedding prevention within student success, wellness, and mental health initiatives [10,11,15,33]. The evidence reviewed here suggests that such institutions function most effectively as protective ecosystems when prevention is linked to belonging and development rather than to surveillance alone [11,15,33,35]. This has practical implications for campus and school leaders, who are often tasked with responding to substance-related concerns through policy and compliance mechanisms but may undervalue the preventive role of engagement, support, and inclusive climate. A resilience-informed model suggests that educational prevention should not only communicate risk, but also cultivate the conditions under which healthier choices become more sustainable and more developmentally coherent [10,11,15,35]. For adolescence and young adulthood alike, the educational environment may therefore be one of the most strategic sites for strengthening resilience as a public health resource [11,15,33].

The discussion would be incomplete without attention to equity. One of the clearest themes in the literature is that resilience cannot be understood responsibly apart from structural conditions and cultural context [14,20,36,37,38,39,40,41]. Trauma exposure, social marginalization, racism, stigma, geographic isolation, and limited institutional access can all constrain the development or mobilization of protective processes, even in youth who possess considerable internal strengths [20,36,40,41]. This means that resilience-based prevention must avoid sliding into a rhetoric of personal responsibility that implicitly blames individuals for failing to overcome conditions that are socially produced [20,22,36]. Instead, the most ethically robust use of resilience in prevention is one that recognizes both agency and inequality, and that views support, access, and cultural relevance as essential components of protection rather than as optional enhancements [20,36,37,38,39,40]. This perspective is especially important for diverse and underserved populations, for whom culturally grounded and community-informed prevention may be central to whether resilience-based interventions are experienced as meaningful and effective [14,37,38,39].

At the same time, a critical reading must acknowledge tensions that the resilience framework does not fully resolve. Conceptually, the same breadth that makes resilience integrative can make it difficult to falsify: a framework able to accommodate almost any positive factor risks explaining outcomes after the fact rather than predicting them, which is why the analytic separation of resilience from its protective factors (Section 3) and the emphasis on prospective evidence (Section 8) function as necessary disciplines rather than technicalities. There are also genuine tensions across levels of explanation. Individual, relational, and structural accounts are not always complementary; foregrounding individual coping can obscure how far outcomes are shaped by structural conditions, whereas foregrounding structure can understate the real variation in adaptation among young people facing similar circumstances. Multilevel framing is often invoked as a resolution, but it can itself become a way of deferring the harder question of which level carries the causal work in a given case, and current evidence rarely partitions effects cleanly across levels. Recognizing these tensions does not diminish the value of resilience as an organizing lens, but it does caution against treating the multilevel, ecological account as a settled empirical fact rather than as a still-developing and only partially tested interpretation.

Taken together, the literature reviewed in this article suggests that resilience has genuine value for substance use prevention, but that its usefulness depends on how carefully it is conceptualized and applied [6,7,19,20,22,23,24]. Resilience is most compelling when it is treated as a multilevel process supported by promotive and protective systems, and least compelling when it is reduced to a vague synonym for toughness or success [7,20,22,42,43]. The field has already produced important conceptual and practical insights, yet the variability in definitions, measures, and outcomes shows that additional refinement is still needed [17,18,42,43,44,45]. The next section therefore turns to future directions for research, with particular attention to conceptual clarity, developmental precision, methodological rigor, and the continued integration of resilience with equity-informed public health prevention.

## 10. Future Directions for Research

A first priority for future research is greater conceptual clarity (Table 6 summarizes the key gaps and corresponding priorities discussed in this section). As the present review has shown, resilience continues to be used across the literature in ways that are sometimes overlapping, sometimes inconsistent, and sometimes too broad to be analytically useful [7,21,22,42,43]. Future studies would benefit from specifying more clearly whether resilience is being conceptualized as a process, an outcome, a set of promotive factors, or a broader developmental framework [7,21,22]. This conceptual precision is especially important in substance use prevention because the meaning assigned to resilience shapes the kinds of variables that are measured, the hypotheses that are tested, and the intervention strategies that are prioritized [19,23,24]. Without clearer definitions, the field risks generating findings that appear cumulative while actually referring to different constructs [7,22,42]. More deliberate theoretical framing would therefore strengthen both coherence and comparability across resilience-oriented prevention studies [17,18,21,42].

A second priority is stronger developmental research that captures how resilience-related processes operate across time. Adolescence and young adulthood are not static categories; they are developmental periods marked by transitions in cognitive control, peer salience, family influence, institutional attachment, and adult role formation [3,4,5,25,26,27,28]. Future work should therefore examine how protective processes shift across these periods, whether certain factors are more influential at particular developmental stages, and how early protective experiences may shape later substance use trajectories [3,15,16,24,28]. Longitudinal designs are especially important because they offer stronger leverage for understanding temporal order, developmental timing, and mechanisms of change than cross-sectional studies can provide [15,16,22]. Such work could also help clarify whether resilience operates differently for delaying initiation, reducing escalation, interrupting harmful coping, or supporting later desistance from use [3,28]. Greater developmental precision would allow resilience-informed prevention to become not only more accurate conceptually, but also more targeted in practice [4,24].

A third need is more intervention-focused research that identifies which resilience-building components are actually driving preventive effects. The existing literature suggests that multilevel interventions involving schools, families, communities, and youth engagement are promising [29,30,31,32,33,34,35], but more work is needed to determine which elements matter most, for whom, and under what conditions [23,24]. For example, future studies could examine whether improvements in school connectedness, parental communication, coping skills, or cultural identity function as the primary mechanisms linking interventions to substance use outcomes [11,15,29,30,35]. This would allow the field to move beyond broad claims that resilience “matters” and toward a more precise understanding of how prevention programs cultivate protective functioning [19,23,24]. Intervention research would also benefit from more consistent attention to implementation quality, contextual fit, and sustainability, since even well-designed programs may perform differently depending on the settings in which they are delivered [32,35]. In this respect, the future of resilience-informed prevention depends not only on theoretical development, but also on better specification of mechanisms and implementation processes [23,24].

A fourth priority is more inclusive and context-sensitive research. Much remains to be learned about how resilience-based prevention functions across culturally diverse, socially marginalized, rural, and otherwise underserved populations [14,20,36,37,38,39,40,41]. Although the literature increasingly recognizes the importance of structural determinants and culturally grounded approaches, these themes still need deeper integration into mainstream prevention research [20,36,37,38,39,40]. Future studies should examine not only whether interventions are effective, but whether they are culturally resonant, geographically feasible, and responsive to the structural conditions that shape young people’s lives [20,36,39,40]. This is particularly important for populations exposed to trauma, discrimination, chronic stress, or service inequities, where protective processes may take different forms or require different forms of institutional support [20,36,41]. More research grounded in community collaboration and culturally informed theory would substantially strengthen the relevance and equity of resilience-based prevention scholarship [37,38,39]. In this sense, future work should not merely include diverse populations; it should treat diversity and context as central to the theoretical development of the field [20,37].

Finally, future scholarship should advance toward more integrated, multilevel models of prevention. One of the clearest conclusions of the present review is that resilience is not adequately explained by individual variables alone [19,20,24]. Yet much research still isolates personal characteristics from the relational, institutional, and structural environments in which adaptation occurs [20,36]. Future studies would benefit from models that more explicitly examine how internal assets interact with family systems, peer networks, school and campus climates, community structures, and broader social determinants of health [19,20,24,36]. Such integration would move the field closer to a public health understanding of prevention in which protective processes are distributed across systems rather than located only within the individual [1,2,20,23,24]. Ultimately, the most productive next phase of research will ask not simply whether resilience is associated with lower substance use risk, but how, for whom, under what developmental and social conditions, and through which systems resilience most effectively supports substance use prevention [19,20,24].

## 11. Conclusions

Substance use prevention across adolescence and young adulthood benefits from stronger attention to the protective processes that support healthy adaptation under conditions of stress, adversity, and developmental transition [1,3,4,5,6,19,24]. The literature reviewed in this article indicates that resilience offers a valuable framework for this task when it is conceptualized not as a fixed personal trait, but as a multilevel process shaped by internal capacities, supportive relationships, institutional belonging, community resources, and cultural context [6,19,20,22,23,24]. This perspective is particularly important for substance use prevention because it broadens the field beyond deficit-focused models and highlights how positive developmental trajectories are sustained through systems of support rather than through willpower alone [19,20,23,24]. It also aligns closely with public health and health promotion approaches that emphasize upstream conditions, community-centered intervention, and the cultivation of well-being alongside the reduction in risk [1,2,10,23]. In this sense, resilience is useful not because it replaces established prevention science, but because it helps integrate its developmental, relational, and structural dimensions into a more coherent framework [19,24].

At the same time, the present review makes clear that resilience is most meaningful when applied carefully. Its value diminishes when it is used vaguely, measured inconsistently, or detached from the inequities and contexts that shape young people’s opportunities for adaptation [7,20,22,36,42,43,44,45]. The strongest evidence reviewed here suggests that the prevention approaches with the strongest support are those that build not only knowledge and refusal skills, but also connectedness, coping, agency, cultural relevance, and access to supportive systems across family, school, campus, and community settings [9,10,11,12,13,14,15,29,30,31,32,33,34,35,37,38,39]. This is especially important for diverse and underserved populations, for whom resilience may depend as much on equitable access to meaningful resources as on individual strengths [14,20,36,37,38,39,40,41]. A resilience-informed prevention model therefore calls for interventions that are developmental, ecological, and justice-aware. Ultimately, the added value of a resilience-informed approach lies less in reducing exposure to risk alone than in strengthening the conditions under which adolescents and young adults can thrive without turning to substances as a means of coping, belonging, or survival [19,20,23,24].

Two practical priorities follow. For practice, the most defensible first-line investments are family-centered and school-based approaches, which rest on the strongest and most longitudinal evidence, complemented by community-level coordination, as in the Communities That Care model, to extend reach. Campus-based and digital strategies are promising but currently rest on thinner or shorter-term evidence and are therefore best deployed as rigorously evaluated adjuncts rather than as stand-alone solutions, and equity and cultural grounding should be built into all levels rather than treated as a separate track. For research, the most critical next steps are testable mechanistic questions: whether prospectively strengthening specific protective factors reduces subsequent initiation and escalation, that is, formal tests of resilience as a mediating process; whether combined, multilevel interventions outperform single-level ones; whether protective mechanisms differ between adolescence and young adulthood and between college and non-college populations; whether buffering effects are reliably detectable under high adversity; and whether reductions in substance use themselves feed back to restore protective factors. Prioritizing these questions would move the field from asserting that resilience matters toward specifying how, for whom, and under what conditions it does.

Finally, two threads in this literature deserve emphasis because they reinforce the integrative view advanced here. First, resilience is shaped by early developmental history: adverse childhood experiences and other early adversity are associated not only with earlier substance involvement but with enduring patterns of stress response, emotion regulation, and self-concept that condition how later protective resources are recognized and used [22,24,41]. Understood this way, resilience is partly rooted in the cognitive and emotional organization laid down by early experience, which is why trauma-informed and relationally supportive prevention is important rather than incidental. Second, adolescent substance use is embedded in emotional and relational dynamics, often functioning as a means of managing distress, regulating affect, or negotiating belonging and social position, and it frequently co-occurs with other emotionally driven behavioral risks [5,10,19,24,34]. Prevention that engages only cognitive and behavioral targets, without also strengthening emotion regulation, relational security, and the broader social context, is therefore likely to address only part of the process. Both threads point to the same conclusion: effective prevention is developmental, relational, and emotionally attuned, not merely informational.

## Figures and Tables

**Table 1:** Databases, search terms, and inclusion and exclusion criteria used in the narrative review.

Review Parameter	Specification
Databases searched	PubMed; PsycINFO; Scopus; Web of Science, supplemented by backward and forward citation tracking of key articles and reviews
Core search terms	Combinations of “resilience,” “protective factors,” “substance use prevention,” “addiction prevention,” “health promotion,” “adolescents,” “young adults,” “community prevention,” and “school-based prevention”
Associated constructs	Coping, social connectedness, self-efficacy, and developmental assets, when clearly linked to resilience-oriented prevention
Population focus	Adolescents and young adults, emphasizing prevention before the onset of severe or entrenched substance-related disorder
Inclusion criteria	Peer-reviewed articles and reviews addressing resilience or closely related protective processes relevant to prevention
Exclusion criteria	Studies focused exclusively on treatment, relapse prevention, or recovery, unless offering clear conceptual or practical insight for prevention
Synthesis approach	Thematic synthesis across conceptual, developmental, protective-factor, intervention, population, and methodological domains

**Table 2:** Key conceptualizations of resilience and their relevance to substance use prevention.

Source	Core Conceptualization	Level of Analysis	Relevance to Prevention
Masten [6]	Resilience as “ordinary magic”: positive adaptation arising from normative developmental systems	Individual–developmental	Directs attention to everyday adaptive systems rather than rare individual traits
Rutter [22]	A dynamic, inferential concept shaped by developmental processes and the timing of adversity	Process	Cautions against treating resilience as a static inner strength
Luthar, Cicchetti, and Becker [7]	Positive adaptation within the context of significant adversity; distinct from adjacent constructs	Process–outcome	Stresses conceptual precision and explicit adversity criteria
Windle [21]	Negotiation, adaptation, and management of significant challenge using personal and environmental assets	Individual–environmental	Distinguishes resilience from coping and self-efficacy
Fergus and Zimmerman [19]	Adolescent assets and resources that support healthy development under conditions of risk	Multilevel (ecological)	Shifts focus from individual blame toward promotive factors
Ungar [20]	Social ecology of resilience: navigation toward resources that families, institutions, and communities provide	Social–ecological/cultural	Centers cultural relevance and resource availability
Zimmerman et al. [23,24]	Resiliency theory distinguishing individual assets from environmental resources, emphasizing promotive factors	Multilevel (ecological)	Frames prevention around strengthening promotive processes, not only reducing risk

**Table 3:** Developmental contrasts between adolescence and young adulthood relevant to resilience-based prevention.

Dimension	Adolescence	Young Adulthood
Core developmental features	Heightened reward and novelty sensitivity, identity formation, and still-maturing self-regulation [25,26]	Identity exploration, instability, self-focus, and transitions in work, education, and relationships [4]
Salient risk processes	Peer reorientation and the increasing salience of peer norms surrounding substances [5,25,27]	Greater autonomy with reduced social control and exposure to novel environments [3,4]
Where resilience is primarily anchored	Family support, school climate, external structure, and access to caring adults [9,11,12,13,19,24]	Internal self-regulation and goal orientation integrated with peer networks, institutional belonging, and role commitments [3,4,15,16,24,28]
Prevention implication	Early intervention during a period of high plasticity and social sensitivity [5,25,26]	Support through transition, including “maturing out” of heavier use as adult roles emerge [5,28]

**Table 4:** Resilience-related protective factors and candidate protective mechanisms associated with lower substance use risk across ecological levels.

Ecological Level	Representative Protective Factors	Example Mechanism	Key Sources
Individual	Emotional regulation, adaptive coping, self-efficacy, problem-solving, future orientation, refusal skills, optimism, and self-control	Reduces the likelihood that stress or social pressure is managed through substance use	[1,2,19,23,24]
Family	Parental monitoring, warmth and attachment, communication, clear boundaries, and family cohesion	Provides emotional security and behavioral structure that scaffold coping	[1,9,12,13]
Peer/relational	Prosocial peers, supportive friendships, mentoring, belonging, and healthy social norms	Offers alternative sources of identity, status, and support	[5,10,19,27]
School/institutional	School connectedness, engagement, supportive adults, and extracurricular involvement	Strengthens belonging and conventional attachment while reducing alienation	[9,11,15]
Community/cultural	Community cohesion, structured activities, prosocial adults, and cultural identity and continuity	Embeds youth in opportunity structures and culturally meaningful belonging	[12,14,20]

**Table 5:** Representative prevention and health promotion strategies that build resilience.

Strategy and Setting	Representative Approaches	Resilience Component Emphasized	Representative Evidence
School-based	Integrated academic and health education, social-emotional learning, life-skills and coping training, and supportive school climate	School connectedness, engagement, competence, and supportive adult relationships	[11,15,35]
Family-centered	Parent-based programs strengthening communication, monitoring, and rule-setting; combined student- and parent-based programs	Relational structure supporting emotion regulation and behavioral control	[29,30]
Community-based	Community coalitions and coordinated, data-driven prevention systems such as Communities That Care	Collective norms, opportunity structures, and community prevention infrastructure	[20,32]
Campus/young adult	Prevention embedded in student well-being, belonging, help-seeking, and mental health promotion	Belonging, coping, and connection to meaningful roles during transition	[3,4,33]
Digital and technology-mediated	Stand-alone digital psychoeducation, self-monitoring, and coping tools	Scalable reinforcement of coping and self-reflection and improved access	[31]
Integrated mental health	Youth-engaged programs addressing stress, anxiety, depression, trauma, and disconnection alongside substance use	Agency, relevance, and adaptive functioning through meaningful engagement	[10,34]

**Table 6:** Key gaps in the literature and corresponding priorities for future research.

Identified Gap	Why It Matters	Recommendation
Inconsistent conceptualization of resilience	Findings may appear cumulative while actually referring to different constructs [7,22,42]	Specify whether resilience is treated as a process, an outcome, a set of promotive factors, or a broader framework [7,21,22]
Limited developmental and longitudinal evidence	Cross-sectional data constrain inferences about timing, direction, and mechanism [15,16,22]	Use longitudinal designs that trace protective processes across adolescence and young adulthood [3,15,16,28]
Unclear intervention mechanisms	Broad claims that resilience matters obscure which components drive preventive effects [23,24]	Test whether connectedness, parenting, coping, or cultural identity are the active ingredients [11,15,29,30,35]
Insufficient attention to diversity and context	Structural and cultural determinants remain under-integrated into mainstream research [20,36,37,38,39,40]	Center cultural relevance, structural conditions, and community collaboration in design [20,37,38,39]
Overly individualized models	Isolating personal traits ignores the relational and structural ecology of adaptation [19,20,36]	Build multilevel models linking individual assets to family, school, community, and structural factors [19,20,24,36]

## Data Availability

No new data were created or analyzed in this study. Data sharing is not applicable to this article.

## Abbreviations

The following abbreviations are used in this manuscript:

ACEs	Adverse childhood experiences

## References

[1] Hawkins J.D., Catalano R.F., Miller J.Y. (1992). Risk and protective factors for alcohol and other drug problems in adolescence and early adulthood: Implications for substance abuse prevention. Psychol. Bull..

[2] Nawi A.M., Ismail R., Ibrahim F., Hassan M.R., Abdul Manaf M.R., Amit N., Ibrahim N., Shafurdin N.S. (2021). Risk and protective factors of drug abuse among adolescents: A systematic review. BMC Public Health.

[3] Stone A.L., Becker L.G., Huber A.M., Catalano R.F. (2012). Review of risk and protective factors of substance use and problem use in emerging adulthood. Addict. Behav..

[4] Arnett J.J. (2000). Emerging adulthood: A theory of development from the late teens through the twenties. Am. Psychol..

[5] Cousijn J., Luijten M., Feldstein Ewing S.W. (2018). Adolescent resilience to addiction: A social plasticity hypothesis. Lancet Child Adolesc. Health.

[6] Masten A.S. (2001). Ordinary magic: Resilience processes in development. Am. Psychol..

[7] Luthar S.S., Cicchetti D., Becker B. (2000). The construct of resilience: A critical evaluation and guidelines for future work. Child Dev..

[8] Luthar S.S., Cicchetti D. (2000). The construct of resilience: Implications for interventions and social policies. Dev. Psychopathol..

[9] Resnick M.D., Bearman P.S., Blum R.W., Bauman K.E., Harris K.M., Jones J., Tabor J., Beuhring T., Sieving R.E., Shew M. (1997). Protecting adolescents from harm. Findings from the National Longitudinal Study on Adolescent Health. JAMA.

[10] Blum R.W., Lai J., Martinez M., Jessee C. (2022). Adolescent connectedness: Cornerstone for health and wellbeing. BMJ.

[11] Weatherson K.A., O’Neill M., Lau E.Y., Qian W., Leatherdale S.T., Faulkner G.E.J. (2018). The protective effects of school connectedness on substance use and physical activity. J. Adolesc. Health.

[12] Cleveland M.J., Feinberg M.E., Bontempo D.E., Greenberg M.T. (2008). The role of risk and protective factors in substance use across adolescence. J. Adolesc. Health.

[13] Hemphill S.A., Heerde J.A., Herrenkohl T.I., Patton G.C., Toumbourou J.W., Catalano R.F. (2011). Risk and protective factors for adolescent substance use in the United States and Australia: A longitudinal study. J. Adolesc. Health.

[14] Woods C., Kim B., Guo K., Nyguen T., Taplayan S., Aronowitz T. (2022). Factors that influence substance use among American Indian/Alaskan Native youth: A systematic mixed studies review. J. Am. Psychiatr. Nurses Assoc..

[15] Lee H., Henry K.L. (2022). Adolescent substance use prevention: Long-term benefits of school engagement. J. Sch. Health.

[16] Feinberg M.E., Fang S., Fosco G.M., Sloan C.J., Mogle J., Spoth R.L. (2022). Long-term effects of adolescent substance use prevention on participants, partners, and their children: Resiliency and outcomes 15 years later during the COVID-19 pandemic. Prev. Sci..

[17] Ferrari R. (2015). Writing narrative style literature reviews. Med. Writ..

[18] Sukhera J. (2022). Narrative reviews: Flexible, rigorous, and practical. J. Grad. Med. Educ..

[19] Fergus S., Zimmerman M.A. (2005). Adolescent resilience: A framework for understanding healthy development in the face of risk. Annu. Rev. Public Health.

[20] Ungar M. (2011). The social ecology of resilience: Addressing contextual and cultural ambiguity of a nascent construct. Am. J. Orthopsychiatry.

[21] Windle G. (2011). What is resilience? A review and concept analysis. Rev. Clin. Gerontol..

[22] Rutter M. (2012). Resilience as a dynamic concept. Dev. Psychopathol..

[23] Zimmerman M.A. (2013). Resiliency theory: A strengths-based approach to research and practice for adolescent health. Health Educ. Behav..

[24] Zimmerman M.A., Stoddard S.A., Eisman A.B., Caldwell C.H., Aiyer S.M., Miller A. (2013). Adolescent resilience: Promotive factors that inform prevention. Child Dev. Perspect..

[25] Steinberg L. (2008). A social neuroscience perspective on adolescent risk-taking. Dev. Rev..

[26] Dahl R.E. (2004). Adolescent brain development: A period of vulnerabilities and opportunities. Keynote address. Ann. N. Y. Acad. Sci..

[27] Nelson E.E., Leibenluft E., McClure E.B., Pine D.S. (2005). The social re-orientation of adolescence: A neuroscience perspective on the process and its relation to psychopathology. Psychol. Med..

[28] Windle M. (2020). Maturing out of alcohol use in young adulthood: Latent class growth trajectories and concurrent young adult correlates. Alcohol. Clin. Exp. Res..

[29] Kuntsche S., Kuntsche E. (2016). Parent-based interventions for preventing or reducing adolescent substance use: A systematic literature review. Clin. Psychol. Rev..

[30] Newton N.C., Champion K.E., Slade T., Chapman C., Stapinski L., Koning I., Tonks Z., Teesson M. (2017). A systematic review of combined student- and parent-based programs to prevent alcohol and other drug use among adolescents. Drug Alcohol Rev..

[31] Aneni K., Meyer J., Funaro M., Pegram D., Umutoni F., Gomati de la Vega I., Jiao M., Fernandes C., Onyeaka H., Baiden P. (2023). A systematic review and meta-analysis evaluating the efficacy of stand-alone digital interventions to prevent substance use among adolescents. Curr. Addict. Rep..

[32] Van Horn M.L., Fagan A.A., Hawkins J.D., Oesterle S. (2014). Effects of the Communities That Care system on cross-sectional profiles of adolescent substance use and delinquency. Am. J. Prev. Med..

[33] Dennhardt A.A., Murphy J.G. (2013). Prevention and treatment of college student drug use: A review of the literature. Addict. Behav..

[34] Dunne T., Bishop L.D., Avery S.J., Darcy S.J. (2017). A review of effective youth engagement strategies for mental health and substance use interventions. J. Adolesc. Health.

[35] Melendez-Torres G.J., Tancred T., Fletcher A., Thomas J., Campbell R., Bonell C. (2018). Does integrated academic and health education prevent substance use? Systematic review and meta-analyses. Child Care Health Dev..

[36] Cunningham J.K., Saleh A.A. (2024). Structural stigma, racism, and sexism studies on substance use and mental health: A review of measures and designs. Alcohol Res..

[37] Lauricella M., Valdez J.K., Okamoto S.K., Helm S., Zaremba C. (2016). Culturally grounded prevention for minority youth populations: A systematic review of the literature. J. Prim. Prev..

[38] Hecht M.L., Marsiglia F.F., Elek E., Wagstaff D.A., Kulis S., Dustman P., Miller-Day M. (2003). Culturally grounded substance use prevention: An evaluation of the keepin’ it *R.E.A.L.* curriculum. Prev. Sci..

[39] Okamoto S.K., Helm S., Pel S., McClain L.L., Hill A.P., Hayashida J.K.P. (2014). Developing empirically based, culturally grounded drug prevention interventions for Indigenous youth populations. J. Behav. Health Serv. Res..

[40] Rhew I.C., Hawkins J.D., Oesterle S. (2011). Drug use and risk among youth in different rural contexts. Health Place.

[41] Dube S.R., Miller J.W., Brown D.W., Giles W.H., Felitti V.J., Dong M., Anda R.F. (2006). Adverse childhood experiences and the association with ever using alcohol and initiating alcohol use during adolescence. J. Adolesc. Health.

[42] Windle G., Bennett K.M., Noyes J. (2011). A methodological review of resilience measurement scales. Health Qual. Life Outcomes.

[43] Ballard M., Gill P.R., Hand T., MacKenzie D. (2024). A critical evaluation of adolescent resilience self-report scales: A scoping review. Child. Youth Serv. Rev..

[44] Winters K.C., Stinchfield R.D., Henly G.A., Schwartz R.H. (1990). Validity of adolescent self-report of alcohol and other drug involvement. Int. J. Addctn..

[45] Harris K.M., Griffin B.A., McCaffrey D.F., Morral A.R. (2008). Inconsistencies in self-reported drug use by adolescents in substance abuse treatment: Implications for outcome and performance measurements. J. Subst. Abus. Treat..

